# Resveratrol Sensitizes Tamoxifen in Antiestrogen-Resistant Breast Cancer Cells with Epithelial-Mesenchymal Transition Features

**DOI:** 10.3390/ijms140815655

**Published:** 2013-07-26

**Authors:** Xiao-Peng Shi, Shan Miao, Yin Wu, Wei Zhang, Xiao-Fang Zhang, Hua-Zhao Ma, Hai-Li Xin, Juan Feng, Ai-Dong Wen, Yan Li

**Affiliations:** 1 Department of Pharmacy, Xijing Hospital, Fourth Military Medical University, Xi’an 710032, China; E-Mails: shixiaop@fmmu.edu.cn (X.P.S.); wuyin_2005@126.com (Y.W.); zxf119bme403@163.com (X.-F.Z.); feng.pharmacy@gmail.com (J.F.); 2 Institute of Materia Medica, Fourth Military Medical University, Xi’an 710032, China; E-Mail: miaoshan@fmmu.edu.cn; 3 School of Public Health, Fourth Military Medical University, Xi’an 710032, China; E-Mail: zhanghanyu887@163.com; 4 Outpatient Department, Institute of Armored Force, Bengbu 233050, China; E-Mail: ahbbzjbxymhc@163.com; 5 Surgery Pharmacy of Department of Pharceutical Care, General Hospital of PLA, Beijing 100853, China; E-Mail: xiaoa63@163.com

**Keywords:** breast cancer, tamoxifen, resistance, epithelial-mesenchymal transition, resveratrol

## Abstract

Tamoxifen resistance remains to be a huge obstacle in the treatment of hormone-dependent breast cancer, and this therefore highlights the dire need to explore the underlying mechanisms. The epithelial-mesenchymal transition (EMT) is a molecular process through which an epithelial cell transfers into a mesenchymal phenotype. Roles of EMT in embryo development, cancer invasion and metastasis have been extensively reported. Herein, we established tamoxifen-resistant MCF-7/TR breast cancer cells and showed that MCF-7/TR cells underwent EMT driven by enhanced endogenous TGF-β/Smad signaling. Ectopic supplement of TGF-β promoted in MCF-7 cells a mesenchymal and resistant phenotype. In parallel, we demonstrated that resveratrol was capable of synergizing with tamoxifen and triggering apoptosis in MCF-7/TR cells. Further Western blot analysis indicated that the chemosensitizing effects of resveratrol were conferred with its modulation on endogenous TGF-β production and Smad phosphorylation. In particular, 50 μM resveratrol had minor effects on MCF-7/TR cell proliferation, but could significantly attenuate endogenous TGF-β production and the Smad pathway, ultimately leading to reversion of EMT. Collectively, our study highlighted distinct roles of EMT in tamoxifen resistance and resveratrol as a potential agent to overcome acquired tamoxifen resistance. The molecular mechanism of resveratrol chemosensitizing effects is, at least in part, TGF-β/Smad-dependent.

## 1. Introduction

Breast cancer remains the most prevalent cancer in females worldwide and ranks as the second cause of cancer-related deaths. Nearly 65% of breast cancers are hormone-dependent and require antiestrogen therapy [1]. Tamoxifen is a selective estrogen receptor (ER) modifier that competitively inhibits the interaction between estrogen and ER. Tamoxifen is predominantly administered as first-line treatment for both early and advanced ER-positive breast cancer patients. Preclinical studies have shown that tamoxifen treatment can induce breast cancer cell growth arrest and death, which are consistent with the clinical efficacy of tamoxifen, which can elicit a cessation of tumor growth and increase overall survival. Nonetheless, approximately 50% of breast cancer patients who initially respond well ultimately developed unresponsiveness and relapse in the clinical settings of continuous tamoxifen exposure [2,3], thus highlighting the dire need to probe the underlying molecular mechanisms and to identify corresponding novel therapeutics to overcome acquired tamoxifen resistance.

An increasing number of studies have indicated that multiple mechanisms are involved in this process [4], including ER heterogeneity and mutation, mitogenic growth factor production and abnormal expression of growth factor receptors [5–7]. Numerous therapeutic strategies targeting related molecules have been discovered and are currently under evaluation [8,9]. However, acquired resistance to tamoxifen may likewise correlate with other novel mechanisms.

In recent years, the importance of an epithelial to mesenchymal phenotypic switch in cancer cells (also termed as epithelial-mesenchymal transition (EMT)) has been extensively documented. EMT is associated with the gain of aggressive characteristics and the progression in various kinds of cancers [10]. EMT is a molecular program, whereby epithelial cells undergo reprogramming from a polarized, differentiated phenotype with numerous cell-cell junctions to acquire a mesenchymal phenotype. It is characterized by the loss of the epithelial phenotype markers, E-cadherin and β-catenin, and the gain of the mesenchymal molecules, vimentin and N-cadherin, disruption of cell polarity, remodeling of cytoskeleton and cell migration [11]. Acquisition of an EMT phenotype allows cancer cells to infiltrate surrounding tissues, as well as distant metastasis. To date, emerging data highlight that EMT in cancer cells is associated with increased tolerance to conditions that could trigger apoptotic cell death [12]. Several clinical studies have shown that elevated expression of the epithelial adhesion molecule, E-cadherin, is associated with favorable survival in colon and esophageal cancers, whereas it is likewise documented that EMT in chemo- and radio-resistant lung and pancreatic cancer cells correlated with a decreased expression of E-cadherin and an increase in the expression of vimentin [13,14]. Additionally, restoring E-cadherin expression or reversing EMT in resistant cancer cells would enable cells to be susceptible to chemotherapy and radiotherapy [15,16]. Consistent with these studies, breast cancer cells displaying the mesenchymal phenotype are relatively resistant to chemotherapeutic agents compared to the epithelial phenotype [17,18]. Taken together, therapeutic strategies that connect EMT to drug might be a novel approach to overcome acquired tamoxifen resistance in breast cancer.

The beneficial effects of diet on cancer progression are gathering considerable interest nowadays. Among various dietary factors, resveratrol (3,5,4-trihydroxy-trans-stilbene), a phenolic phytoalexin component extracted from grapes, berries and peanuts, has a broad spectrum of biological effects and exhibited great merits for public health [19]. Particularly, resveratrol and its derivatives have been regarded as anti-cancer agents against different cancer cell types [20–23]. Although several studies indicated that resveratrol was capable of directly inducing apoptosis and acting as a chemosensitizer in breast cancer cells [24–26], there are no comprehensive investigations hitherto examining the potential effects of resveratrol on tamoxifen-resistant breast cancer cells with EMT characteristics.

In the present study, we established a tamoxifen-resistant breast cancer cell line and provided preliminary evidence suggesting that acquired tamoxifen resistance is closely associated with EMT. We also showed that loss of the epithelial phenotype contributes to decreased tamoxifen susceptibility. Importantly, our study demonstrated that resveratrol at non-cytotoxic concentrations can synergize with tamoxifen and restore drug sensitivity, and this effect may be partially dependent on the regulation of EMT. Taken together, we revealed a molecular mechanism that may serve as a novel target for the treatment of resistant breast cancer.

## 2. Results and Discussion

### 2.1. Results

#### 2.1.1. Acquired Tamoxifen Resistant Breast Cancer Cells Undergo EMT

**Figure 1:**
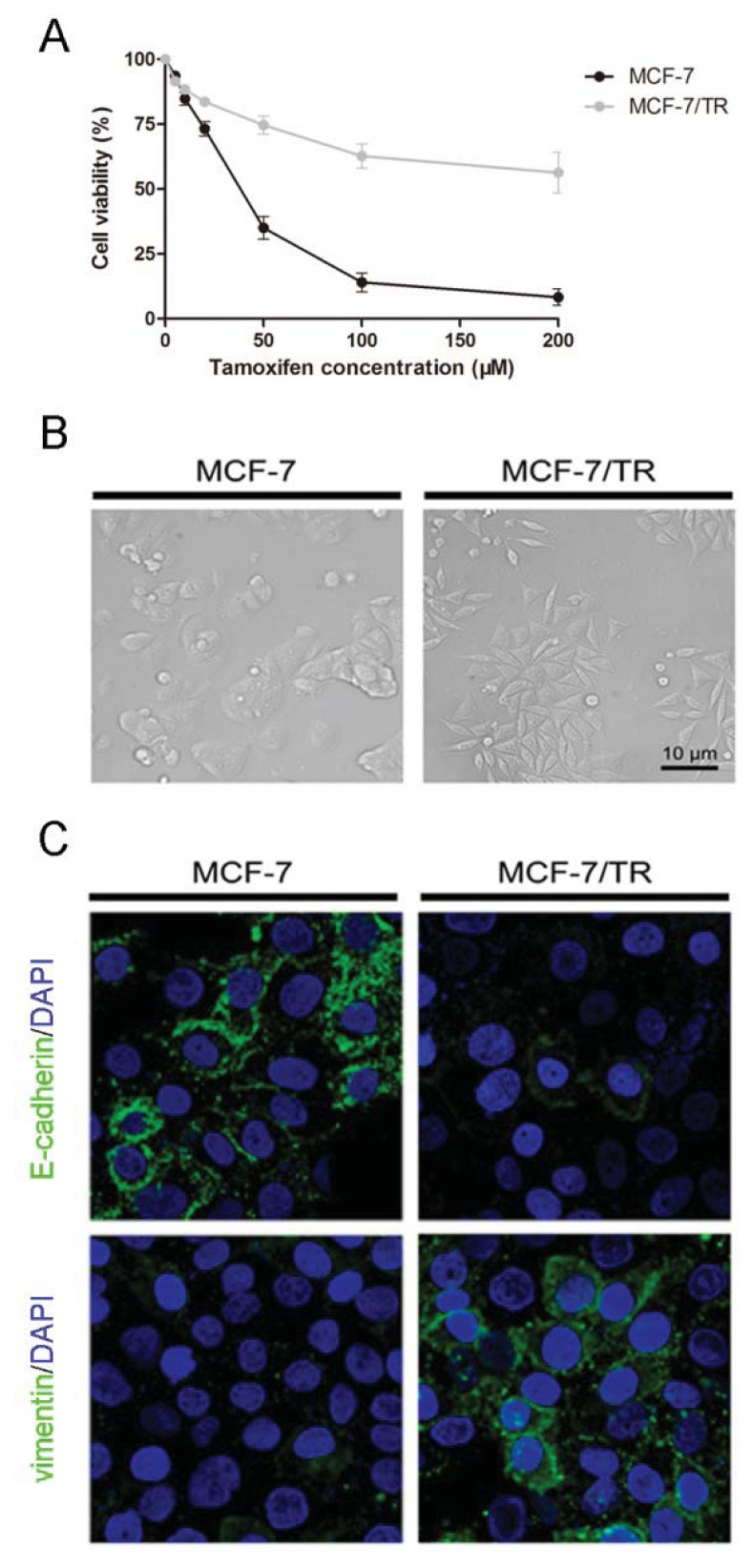
Evidence of epithelial-mesenchymal transition (EMT) in breast cancer tamoxifen-resistant MCF-7/TR cells. (**A**) In comparison with the parental MCF-7 cells, methyl thiazolyl tetrazolium (MTT) assay showed that the daughter MCF-7/TR cells were resistant to tamoxifen treatment; (**B**) when observed under microscope at 200× magnification, parental MCF-7 cells displayed an epithelioid and cobblestone appearance. In contrast, the phenotypic changes observed in resistant MCF-7/TR cells included loss of cell polarity, causing a spindle-shaped morphology and increased formation of pseudopodia. Scale bar = 10 μm; (**C**) immunofluorescent cytochemistry and confocal analysis showed that E-cadherin was markedly expressed in MCF-7 cells, while MCF-7/TR cells expressed vimentin.

In an effort to generate the daughter MCF-7/TR cells, the parental epithelial phenotype breast cancer MCF-7 cell line was continuously exposed to stepwise increasing concentrations of tamoxifen and maintained in 50 μM tamoxifen for 10 months. To test whether the daughter MCF-7/TR cells were resistant to tamoxifen, parental MCF-7 and daughter MCF-7/TR cells were treated with different concentrations of tamoxifen for 48 h. Methyl thiazolyl tetrazolium (MTT) study revealed the MCF-7/TR cell viabilities in the presence of 50, 100 and 200 μM tamoxifen were markedly higher than MCF-7 cells, suggesting a stable resistance in MCF-7/TR cells (Figure 1A). When the morphology of MCF-7 and MCF-7/TR cells was investigated, we observed that MCF-7 cells displayed a cobblestone-like appearance and tight cell-cell junctions. The shape of marginal cells was rounded, exhibiting little formation of pseudopodia. By contrast, MCF-7/TR cells revealed the loss of cell-to-cell junctions, increased formation of pseudopodia and spindle shaped mesenchymal-like morphology (Figure 1B).

Immunofluorescent cytochemistry was thus employed to confirm whether MCF-7/TR cells obtained mesenchymal features (Figure 1C). By using confocal laser scanning microscopy, we found that the epithelial phenotype marker, E-cadherin, was markedly expressed and localized in the membranes of MCF-7 cells, whereas E-cadherin in MCF-7/TR cells was barely detectable. The mesenchymal molecule, vimentin, was weakly distributed in a punctate manner in parental MCF-7 cells, but was significantly induced in the cytoplasm of MCF-7/TR cells.

**Figure 2:**
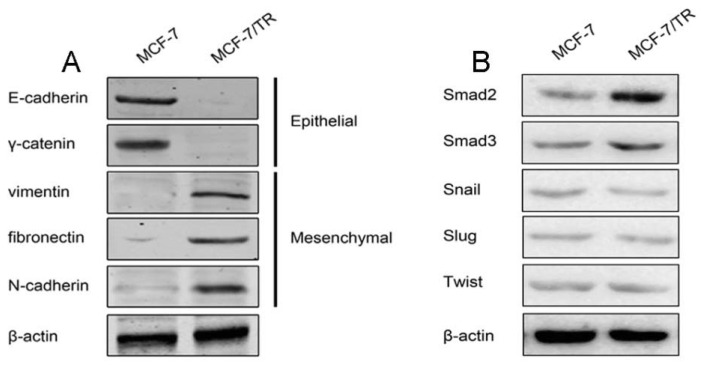
Western blot analysis of EMT biomarkers and transcription factors. (**A**) Western blot demonstrated that MCF-7 cells predominantly expressed epithelial phenotype markers, but MCF-7/TR cells predominantly expressed mesenchymal markers; (**B**) western blot analysis of EMT transcription factors, Smad, Snail, Slung and Twist, in MCF-7 and MCF-7/TR cells.

Consistent with the results from morphological and confocal study, Western blot analysis for EMT markers also revealed that MCF-7/TR cells harbored EMT features (Figure 2A). Epithelial phenotype markers, E-cadherin and γ-catenin, were predominantly expressed in MCF-7 cells, while mesenchymal phenotype-associated molecules, vimentin, fibronectin and N-cadherin, were all upregulated in MCF-7/TR cells.

Smad, Snail, Slug and Twist are four well-documented EMT regulatory transcription factors. By means of Western blot, we found that Snail, Slug and Twist expressions were marginally changed and might not be involved in the mesenchymal phenotype of MCF-7/TR cells, while Smad was predominantly overexpressed in MCF-7/TR cells (Figure 2B).

#### 2.1.2. TGF-β Triggers EMT and Confers Tamoxifen Resistance

**Figure 3:**
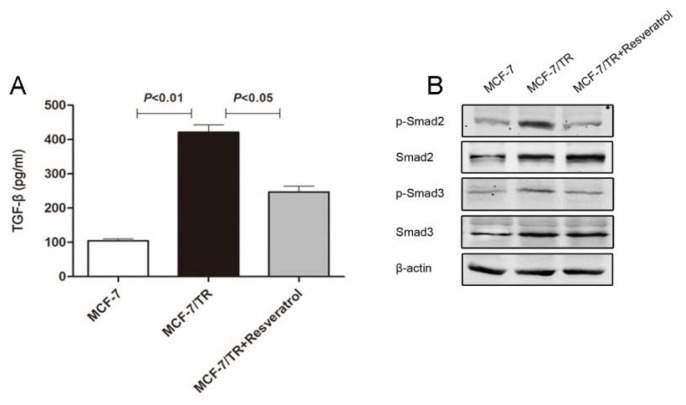
Measurement of TGF-β and Smad signaling in MCF-7 and MCF-7/TR cells. (**A**) The production of endogenous TGF-β in the supernatant of MCF-7 and MCF-7/TR cells without or with resveratrol treatment were measured by ELISA. Data are expressed as the mean ± SEM from three independent experiments performed in triplicate. *p* < 0.01 *vs*. MCF-7 cell and *p* < 0.05 *vs*. resveratrol treatment; (**B**) Baseline expressions of p-Smad2, Smad2, p-Smad3 and Smad3 in MCF-7 and MCF-7/TR cells. Upon resveratrol treatment, the Smad cascade was inhibited in MCF-7/TR cells. β-actin was employed as equal loading.

As we have demonstrated that MCF-7/TR cells underwent EMT with elevated Smad expressions, we subsequently evaluated whether EMT directly conferred tamoxifen resistance. Considering that TGF-β is produced in an autocrine/paracrine manner and can activate Smad signaling in the development of EMT [27], soluble TGF-β from parental MCF-7 and resistant MCF-7/TR cells were measured. Baseline levels of soluble TGF-β were detected in MCF-7 cell culture supernatant, with much higher levels in the MCF-7/TR cell culture supernatant (four-fold *vs.* MCF-7 cells, *p* < 0.01) (Figure 3A). As expected, intracellular Smad2 and Smad3 phosphorylation and the TGF-β/Smad signaling cascade were much more enhanced in MCF-7/TR cells compared to that in MCF-7 cell (Figure 3B). These results demonstrated abnormal production of endogenous TGF-β, and enhanced activation of the TGF-β/Smad pathway could trigger EMT, especially in MCF-7/TR cells.

**Figure 4:**
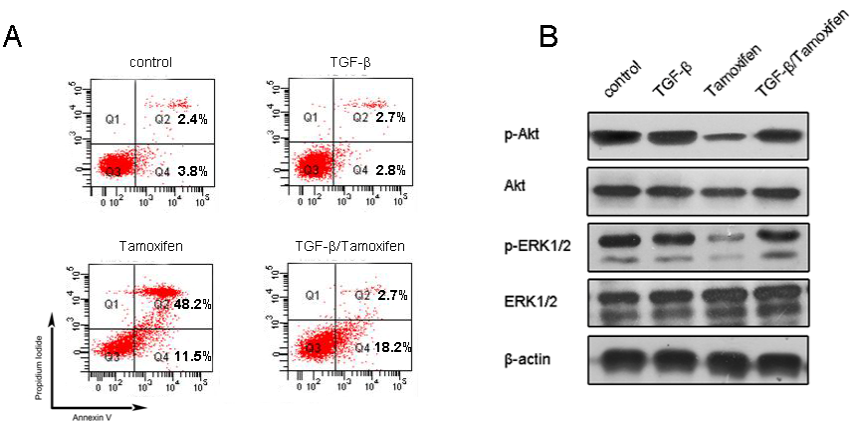
Effects of TGF-β on tamoxifen sensitivity. (**A**) The cancer cells undergoing apoptosis were quantitatively determined by flow cytometry; (**B**) Akt and ERK1/2 phosphorylation status in tamoxifen-treated MCF-7 cells, with or without TGF-β pretreatment.

To further explore whether TGF-β-induced EMT was able to affect tamoxifen sensitivity, the parental tamoxifen sensitive MCF-7 cells were pretreated with 5 ng/mL ectopic TGF-β for 24 h, followed by 50 μM tamoxifen. Flow cytometry analysis showed that 5 ng/mL of TGF-β single agent treatment did not cause MCF-7 cells apoptosis. Fifty micrometers of tamoxifen treatment caused extensive apoptotic cell death, including 11.5% early apoptosis and 48.2% late apoptosis, respectively. Though there was a slight increase in the early apoptosis ratio (18.2% *vs.* 11.5%), TGF-β pretreatment attenuated tamoxifen’s cytotoxic effect and decreased the apoptosis ratio to 20.9% (Figure 4A). Additionally, TGF-β alone did not affect Akt/ERK signaling, while tamoxifen treatment abrogated Akt and ERK1/2 phosphorylation. It was noted that TGF-β pretreatment abolished these actions of tamoxifen in parental MCF-7 cells (Figure 4B). To this end, it is tempting to speculate that apart from inducing EMT, autocrine/paracrine TGF-β and its downstream Smad signaling might play a survival role in breast cancer cells and lead to acquired tamoxifen resistance.

#### 2.1.3. Effects of Resveratrol on MCF-7 and MCF-7/TR Cell Proliferation

**Figure 5:**
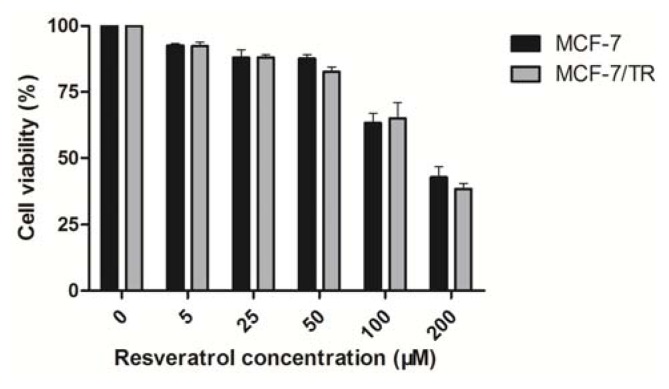
Effects of 48 h resveratrol treatment on MCF-7 and MCF-7/TR cell viability measured by MTT assay. Data are expressed as the mean ± SEM from three independent experiments performed in triplicate.

Preliminary dose response experiments determined that the 50% inhibitory concentration of resveratrol on parental MCF-7 and resistant MCF-7/TR cells was approximately 146.3 μM, with less than 20% inhibition at concentrations of 50 μM (Figure 5; no significant difference between MCF-7 and MCF-7/TR cells). This 50 μM dose is very close to the plasma levels achieved with daily dietary intake of resveratrol. Thus, all subsequent experiments will be conducted at this concentration (0~50 μM) to investigate the effects of resveratrol on tamoxifen sensitivity in MCF-7/TR cells.

#### 2.1.4. Resveratrol Sensitizes MCF-7/TR Cells to Tamoxifen and Promotes Apoptosis

**Figure 6:**
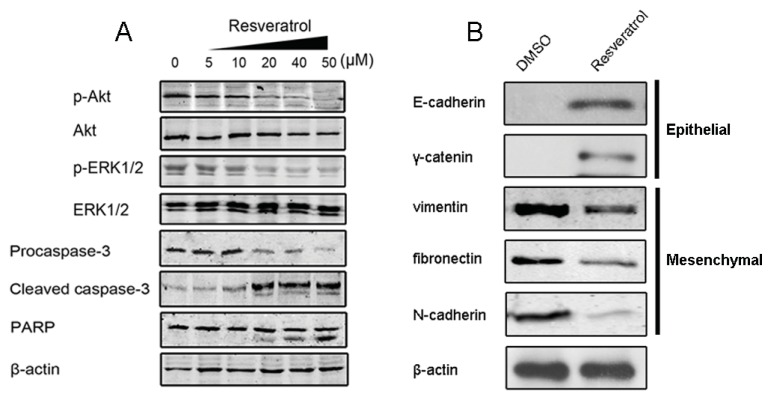
Resveratrol reverses EMT and triggers MCF-7/TR cell apoptosis. Western blot showed (**A**) 50 μM tamoxifen co-treatment with resveratrol dose-dependently reduced Akt and ERK1/2 phosphorylation, promoted caspase-3 activation and polyADP-ribosepolymerase (PARP) cleavage; (**B**) Resveratrol also restored epithelial marker expressions and reversed EMT in MCF-7/TR cells. β-actin was employed as equal loading.

Previous studies have documented that activation of the PI3K/Akt and ERK1/2 signaling pathways plays a critical role in cancer cell survival and resistance [28], and therefore, targeting these pathways may serve as potential therapeutic strategies. We thus explore whether resveratrol at non-cytotoxic concentrations (less than 50 μM) regulates these pathways and synergizes with tamoxifen in resistant breast cancer cells. MCF-7/TR cells were treated with 50 μM tamoxifen without (0 μM) or with various doses of resveratrol (5, 10, 20, 40 and 50 μM, respectively). After the treatment of 48 h, Western blot analysis showed that p-Akt and p-ERK1/2 in MCF-7/TR cells were evident, though in the presence of tamoxifen (50 μM). However, treatment of tamoxifen in combination with resveratrol resulted in a remarkable inhibition of Akt and ERK1/2 phosphorylation, especially in the 40 μM and 50 μM resveratrol groups. Moreover, co-treatment with two agents also promoted cleavage of caspase-3 and polyADP-ribosepolymerase (PARP) in a dose-dependent manner, suggesting the induction of apoptosis (Figure 6A).

#### 2.1.5. Resveratrol Inhibits TGF-β Production, Reverses EMT and Overcomes Resistance

Since Smad was activated, the production of endogenous TGF-β was enhanced in MCF-7/TR cells and ectopic TGF-β treatment led to tamoxifen resistance in parental MCF-7 cells, we reasoned whether there exists a connection between endogenous TGF-β/Smad-induced EMT and acquired tamoxifen resistance in breast cancer cells. As a result, we found that MCF-7/TR cells co-treated with tamoxifen and resveratrol at a concentration of 50 μM exhibited remarkable apoptosis, while treatment with resveratrol alone at the same concentration exerted no remarkable impact on cell viability. To further identify the underlying mechanism, we examined the effect of resveratrol on endogenous TGF-β production and EMT. The overproduction of TGF-β in MCF-7/TR cells was inhibited by resveratrol treatment (Figure 3A). Furthermore, resveratrol treatment attenuated phosphorylation of endogenous Smad2 and Smad3 (Figure 3B).

On the other hand, resveratrol was capable of restoring epithelial markers, while downregulating mesenchymal marker expression in MCF-7/TR cells (Figure 6B). These results highlighted the possibility that resveratrol could reverse EMT and overcome resistance in a TGF-β/Smad-dependent mechanism, therefore affording a potential therapeutic strategy for treatment of breast cancer.

### 2.2. Discussion

In this study, by utilizing an *in vitro* model of tamoxifen resistance, we provided compelling data that endogenous TGF-β/Smad-driven EMT played a critical role in breast cancer cell survival and facilitated the development of acquired tamoxifen resistance. The major novel findings of the present study can be summarized as follows: (1) the development of acquired tamoxifen resistance in MCF-7 cells is closely associated with EMT; (2) overproduction of endogenous TGF-β and its downstream Smad signaling can drive MCF-7 cells to undergo EMT; (3) resveratrol sensitizes resistant cells to tamoxifen and promotes apoptosis at non-cytotoxic concentrations; (4) the mechanisms for the action of resveratrol are TGF-β/Smad-dependent.

The clinical success of tamoxifen has greatly facilitated the treatment of hormone-dependent breast cancer, but resistance could still develop under certain conditions [29]. Numerous studies have been executed to identify the underlying mechanisms in a variety of laboratory and clinical settings [2,5–10]. Particularly, TGF-β is well known to initiate and maintain EMT, and activation of Smad activity is a key step in TGF-β signal transduction [30]. EMT, which is characterized by loss of cell-to-cell adhesion (specifically, through the dismantling of adherens, tight and gap junctions, as well as loss of cell polarity and increased motility), has been widely studied for its role in embryo development and cancer metastasis, and it can also result in the transformation of a differentiated epithelial cell to a mesenchymal cell [11–13].

Whether EMT contributes to drug resistance is yet to be fully elucidated. Several established resistant cancer cell lines have been documented showing EMT features, suggesting the possibility of the involvement of EMT in the development of drug resistance [31–33]. Herein, we described the distinct role of EMT in the development of drug resistance, using a tamoxifen-resistant breast cancer MCF-7/TR cell line. Contrary to the epithelial morphology of parental MCF-7 cells, we demonstrate that resistant MCF-7/TR cells lose epithelial features and acquire mesenchymal characteristics, including loss of cell-cell adhesive interactions. Although Hiscox *et al*. has reported that tamoxifen resistance in MCF-7 cells promotes EMT-like behavior [18], we have further extended the underlying mechanisms to endogenous TGF-β overproduction and Smad activation. The underlying mechanisms of elevated TGF-β were not fully understood. We speculated that loss of ER-mediated signaling in the presence of tamoxifen might activate alternative signaling pathways that interacted with TGF-β synthesis and promoted endogenous TGF-β release. Our data demonstrate that the cancer cell phenotype switch is at least partially due to an enhanced production of endogenous TGF-β and constitutive activation of Smad signaling. Moreover, our results implied that EMT is indeed involved in a resistant phenotype, because the treatment with exogenous TGF-β also enables MCF-7 cells to gain mesenchymal features and decreased sensitivity to tamoxifen. These results strongly indicate a novel role of EMT and its therapeutic potential to overcome drug resistance.

Given that resveratrol has been proposed to have chemopreventive and chemotherapeutic effects, we subsequently evaluated the significance of this natural phytochemical in overcoming tamoxifen resistance. We firstly recognized that resveratrol could cause a cytotoxic effect with an IC_50_ value of ~146.3 μM, while 50 μM resveratrol only caused less than 20% inhibition in both MCF-7 and MCF-7/TR cells, indicating that resveratrol has a minor effect on breast cancer cell viability at concentrations lower than 50 μM. This dose is very close to the daily dietary intake of resveratrol. Interestingly, we found that combined resveratrol at non-cytotoxic doses (5, 10, 20, 40 and 50 μM, respectively) with tamoxifen (50 μM) could significantly reduce Akt and ERK1/2 phosphorylation and promote caspase-3 activation and PARP cleavage. Meanwhile, several lines of evidence suggested that resveratrol could influence the synthesis or release of prostaglandin and other cytokines [34,35]; it is possible that resveratrol’s modulation in cytokine activity could elicit TGF-β inhibition, in conjunction with the reduction of Smad phosphorylation, thereby affecting the EMT progression and overcoming drug resistance. This hypothesis is supported by the results that in MCF-7/TR cells, resveratrol treatment suppressed endogenous TGF-β production and the downstream Smad cascade and restored E-cadherin expressions. Taken together, it is likely that resveratrol acts as a chemosensitizer through TGF-β/Smad signaling and EMT inhibition.

## 3. Experimental Section

### 3.1. Cell Culture

ER-positive breast cancer MCF-7 cells were purchased from the American Type Culture Collection (ATCC). The cells were routinely cultured in Dulbecco’s Modified Eagle’s Medium (DMEM) supplemented with 10% fetal bovine serum, 1% l-glutamine, 100 U/mL penicillin, 100 mg/μL streptomycinin, and 10 μg/mL insulin. Tamoxifen-resistant cells (MCF-7/TR) were developed by culturing MCF-7 cells in the presence of progressively increasing concentrations of tamoxifen (5 μM to 200 μM) and, then, maintaining them for over 10 months. Cells were grown in an atmosphere with 5% CO_2_ at 37 °C, seeded in triplicate for all experiments.

### 3.2. Reagents

Resveratrol, tamoxifen, transforming growth factor β (TGF-β), methyl thiazolyl tetrazolium (MTT) and diamidino-phenyl-indole (DAPI) were purchased from Sigma-Aldrich (St. Louis, MO, USA).

### 3.3. Antibodies and Western Blot

Antibodies for immunoblotting include: rabbit anti-caspase-3 monoclonal antibody, rabbit anti-cleaved caspase-3 monoclonal antibody, horseradish peroxidase (HRP)/fluorescein isothiocyanate (FITC) conjugated anti-mouse or anti-rabbit IgG (Santa Cruz Biotechnology, Santa Cruz, CA, USA), rabbit anti-E-cadherin, *N*-cadherin, vimentin, γ-catenin, fibronectin, p-Smad2, Smad2, p-Smad3, Smad3, Snail, Slug, Twist, rabbit anti-p-Akt, Akt, p-ERK1/2, ERK1/2, rabbit anti-PARP polyclonal antibody (Cell Signaling Technology, Danvers, MA, USA) and mouse anti-β-actin monoclonal antibody (Sigma-Aldrich, St. Louis, MO, USA). After indicated treatments, cells were harvested, washed with ice-cold phosphate buffer solution (PBS) and lysed in lysis buffer (20 mM Tris-HCl (pH 7.5), 150 mM NaCl, 5 mM ethylene diamine tetraacetic acid (EDTA) (pH 8)), 1% NP-40, 1 mM NaVO_4_, 20 mM NaPO_4_ (pH 7.6), 10 mM NaF, 3 mM β-glyceroylphosphate and 5 mM sodium pyrophosphate (all from Sigma, St. Louis, MO, USA). Protein (30 μg) was separated by SDS-PAGE, transferred on nitrocellulose membranes, blocked in 5% non-fat milk and incubated with respective primary antibodies (1:1,000 dilution). The membranes were then washed thrice with Tris-buffered saline and Tween 20 (TBST), followed by incubation with HRP-conjugated secondary antibody (1:4,000 dilution) in TBST. Bound antibody was probed using the Enhanced Chemiluminescence System. Chemiluminescent signals were captured using the Fujifilm LAS 3000 system (Fujifilm). All experiments were performed in triplicate.

### 3.4. Cell Viability Assay

The MTT assay was performed to assess the effect of resveratrol on cancer cell viability. Cells were plated in 96-well plates at a density of 5.0 × 10^3^ cells/well and treated with varying concentrations of resveratrol dissolved in dimethyl sulfoxide (DMSO) (final DMSO concentration <0.1%) at different time intervals. Twenty microliters of sterile MTT dye (5 mg/mL) were added at 37 °C for 4 h, then the culture medium was removed, and 150 μL of DMSO was added and thoroughly mixed in for 10 min. Spectrometric absorbance at 570 nm was measured by using a microplate reader. The concentration at which resveratrol produced 50% inhibition of growth was calculated by the relative survival curve.

### 3.5. Immunocytochemistry

MCF-7 and MCF-7/TR cells were plated on cover slips in 24-well plates. Forty-eight hours after cell attachment, the cancer cells were fixed with PBS containing 4% paraformaldehyde and rendered permeable by treatment for 2.5 min with PBS containing 0.1% Triton X-100. The cells were covered with blocking solution (3% bovine serum albumin and 2.5% goat serum in PBS) for 1 h at room temperature and incubated with rabbit anti-E-cadherin or vimentin antibody diluted at 1:200 overnight at 4 °C. These antibodies were stained with goat anti-rabbit secondary antibody conjugated to FITC (Santa Cruz Biotechnology, Santa Cruz, CA, USA) and viewed with a confocal laser scanning microscope (Olympus FV1000, Tokyo, Japan). Cell nuclei were then counterstained using 0.6 μg/mL DAPI. DAPI fluorescent emits light at a wavelength of 358 nm and FITC at 530 nm, respectively.

### 3.6. TGF-β ELISA Analysis

To compare the concentrations of supernatant TGF-β (transforming growth factor beta), MCF-7 and MCF-7/TR cells were subsequently cultured in serum-free culture medium. After 48 h of attachment, the supernatant was collected for endogenous TGF-β measurement. All samples were measured using ELISA, according to the manufacturer’s recommended procedures (Quantikine, R & D Systems, Minneapolis, MN, USA) and run in triplicate. Color intensity was measured at 450 nm using a spectrophotometric plate reader. TGF-β concentrations were determined by comparison with standard curves.

### 3.7. Apoptosis and Flow Cytometric Studies

Apoptosis was assessed by Annexin V-FITC and propidium iodide (PI) dual staining (BD Biosciences, San Diego, CA, USA). Briefly, cells in each group were collected and washed twice with 0.01 M PBS and suspended in 200 μL binding buffer and, then, incubated with 10 μL Annexin-V-FITC and 5 μL PI for 30 min at 4 °C. The cells undergoing apoptosis were detected by flow cytometry (Beckman Coulter, Brea, CA, USA). At least 10^4^ cells were analyzed for each determination.

### 3.8. Statistical Analysis

Experiments presented in the figures and tables are representative of three or more different repetitions. Values are expressed as the mean ± SD. Clampfit software and ORIGIN6.1 software were used to analyze the data. One-way analysis of variance (ANOVA) was employed to determine the statistical significance between different groups. Significant difference was set at *p* < 0.05.

## 4. Conclusions

Our results indicated a key survival role for endogenous TGF-β/Smad signaling and EMT that facilitates the progression of a breast cancer cell to tamoxifen resistance. To the best of our knowledge, our studies are the first report linking endogenous TGF-β production, the Smad pathway and EMT in the development of tamoxifen resistance. The effects of resveratrol can modulate TGF-β-dependent EMT and, in return, resensitize resistant cells to tamoxifen. Our and others’ studies specified that EMT is linked to chemotherapy resistance, and targeting EMT may provide new approaches during anticancer therapy. More specifically, we propose that resveratrol can reverse EMT in breast cancer cells undergoing tamoxifen resistance.
